# Morphology, Ultrastructure, and Mitochondrial Genome of the Marine Non-Photosynthetic Bicosoecid *Cafileria marina* Gen. et sp. nov.

**DOI:** 10.3390/microorganisms7080240

**Published:** 2019-08-05

**Authors:** Dagmar Jirsová, Zoltán Füssy, Jitka Richtová, Ansgar Gruber, Miroslav Oborník

**Affiliations:** 1 Institute of Parasitology, Biology Centre, Czech Academy of Sciences, Branišovská 31, 370 05 České Budějovice, Czech Republic; 2 Faculty of Science, University of South Bohemia, Branišovská 31, 370 05 České Budějovice, Czech Republic

**Keywords:** heterotrophic nano-flagellate, heterokonta, bicosoecida, phylogeny, flagellar apparatus, new genus

## Abstract

In this paper, we describe a novel bacteriophagous biflagellate, *Cafileria marina* with two smooth flagellae, isolated from material collected from a rock surface in the Kvernesfjorden (Norway). This flagellate was characterized by scanning and transmission electron microscopy, fluorescence, and light microscopy. The sequence of the small subunit ribosomal RNA gene (18S) was used as a molecular marker for determining the phylogenetic position of this organism. Apart from the nuclear ribosomal gene, the whole mitochondrial genome was sequenced, assembled, and annotated. Morphological observations show that the newly described flagellate shares key ultrastructural characters with representatives of the family Bicosoecida (Heterokonta). Intriguingly, mitochondria of *C. marina* frequently associate with its nucleus through an electron-dense disc at the boundary of the two compartments. The function of this association remains unclear. Phylogenetic analyses corroborate the morphological data and place *C. marina* with other sequence data of representatives from the family Bicosoecida. We describe *C. marina* as a new species from a new genus in this family.

## 1. Introduction

Heterotrophic flagellates are one of the cornerstones of aquatic ecosystems, as they are the prime bacterial consumers in the microbial food web and they are among the most important nutrient remineralizers and intermediators to higher levels of the trophic cascade [1,2]. These organisms inhabit a variety of ecosystems, including low-level oxygen or anoxic bottom waters [3,4,5], deep-sea sediments [6], and hypersaline environments [7]. Free-living heterotrophic and mixotrophic stramenopiles represent an important part of pelagic food webs, due to their capability to consume bacteria or dissolved organic material. Moreover, in some habitats, they represent about one third of all heterotrophic flagellates and thus, majorly contribute to a big part of nutrient recycling in the environment [8,9].

Stramenopiles are a highly variable group ranging from heterotrophic nanoprotists to giant phototrophic multicellular kelps [10,11,12]. Stramenopila can be divided into obligate heterotrophs, the apparently plastid-less labyrinthulomycetes, oomycetes, opalinids, and bicosoecids [10,11]; a plastid-containing group, mostly phototrophic Ochrophyta [13,14], that also includes known non-photosynthetic species such as apochlorotic nonphagotrophic diatoms from the genera *Nitzschia* [15,16] and *Pseudonitzschia* [15]; and phagotrophic chrysophytes, e.g., *Mallomonas annulate* and *Spumella* sp. [17,18], to name a few. Interestingly, the ochrophytes also retain their plastids when they lose their autotrophic lifestyle [19], while convincing evidence for the presence of a plastid has not been reported for any of the other groups of stramenopiles, which are all non-photosynthetic. Their plastids are complex in origin, and are derived from a rhodophyte ancestor [20,21]. There is quite some debate on the relationship between the different groups of algae in which complex plastids are found (cryptophytes, haptophytes, ochrophytes, and most of the plastid-containing alveolates), as well as on the number and sequence of plastid gains, and losses or transfers between these groups [22,23,24]. Comparisons of the phylogenetic signal found in mitochondrial (mt), plastid, and nuclear genomes, as well as genome/transcriptome-wide targeting predictions, provide insights into this currently unresolved convolute [23,25]. However, these analyses need to be based on representative sets of organisms, covering the true diversity of these phyla. Phylogenomic analyses support the monophyly of the ochrophytes, while the non-photosynthetic stramenopiles consist of Oomycota (which. together with Ochrophyta, form the Gyrista), and Bigyra, a monophyletic group containing all other known heterotrophic stramenopiles [26]. While there are considerable genome sequence data and knowledge on the economically significant Oomycota [27], the other heterotrophic/bacterivorous stramenopiles, e.g., *Platysulcus*, *Pirsonia* and representatives of the MAST-3 clade, are underrepresented in sequence databases and in terms of knowledge about their ecology and physiology, in contrast to their global abundance in aquatic ecosystems [28,29]. Furthermore, Oomycota, due to their mainly saprophytic and parasitic life strategies, and due to their phylogenetic position as part of the Gyrista, should not be considered as the “true” representatives of heterotrophic stramenopiles, especially because many groups in the Bigyra, like species from *Platysulcus* [30], predatoric species of *Developayella*/*Develorapax* [31], and the MAST-4 [32] clade, seem to be exclusively composed of bacteriovorous lineages.

Bicosoecids are heterotrophic flagellates that represent a basal lineage of heterokonts [5,13]. Although this group has been assigned to unicellular photosynthetic stramenopiles due to their ultrastructure [33,34], analyses of molecular data have proven that Bicosoecida (clade Bikosea) are closer to stem stramenopiles [35]. Moreover, Bikosea were assigned to the group Bigyra with *Blastocystis* and Placidozoa as the closely related taxa [26]. The main ultrastructural character distinguishing bicosoecids from other stramenopiles is the architecture of the feeding basket/flagellar apparatus, which was first thoroughly examined in *Cafeteria roenbergensis* and has been established as an identifying feature since that time [30]. Nevertheless, the feeding basket/flagellar apparatus is an ancestral motif, conserved in two different forms in bicosoecids and chromulinalean chrysophytes, but lost in other stramenopiles [35], which lack the flagellar apparatus consisting of microtubular structures. The microtubular root (R3) has not been observed in any other stramenopiles in the sense of forming a prominent loop around the cell and hence it represents the main ultrastructural feature of Bicosoecida.

Due to the limited availability of sequence data from known species, molecular phylogenies are mostly restricted to a small ribosomal subunit of the nuclear RNAs (18S). The 18S rDNA sequences in combination with the feeding basked morphology are suitable as a basis for initial species recognition (see [5,36,37]). To increase the sampling of heterotrophic stramenopiles, we isolated and cultivated a bicosoecid-like nanoflagellate from rock surface material collected in Norway. To determine its phylogenetic position more precisely, we studied the ultrastructure of this isolate and sequenced its mt genome.

## 2. Material and Methods

### 2.1. Culture Conditions

*Cafileria marina* was sampled as part of a rock surface mat community at a 3 m depth off the shore in Gaustad, Norway (Kvernesfjorden fjord, 62°59′07.9″N 7°19′17.4″E). Monoeukaryotic cultures were isolated by dilution and grown in Tissue Culture Flasks with ventilation screw caps in which the bottom was covered with 5–10 mm of liquid f/2 medium [38,39,40], with a salt concentration of 33.3 g/L (Reef salt, Sera, Germany). The following antibiotics were used to clear the culture from ciliates and bacteria in this order: tetracyclin 10 μg/mL; zeocin 10 μg/mL; and ciprofloxacin 5 μg/mL. Cultures were subcultured into the new media with antibiotic every second week. For accelerated growth during culture isolation, the f/2 media were supplemented with Tryptone Soy Broth (TSB) (Oxoid, Thermo-Fisher, Kansas City, KS, USA) (0.2 g/L) and sodium acetate (SA) (2.3 g/L). Media were calibrated to pH 7.9–8.3. Culture flasks with *C. marina* were kept in the dark at 13–16 °C; medium in the culture flasks was changed weekly and fresh cultures were inoculated at bimonthly intervals.

### 2.2. Light and Scanning Electron Microscopy

Light microscopic images were obtained using an Olympus BX53F microscope with Nomarski phase contrast equipped with a cooled digital camera DP72. The cell size measurements were performed using the Sens Standard Image Analysis Software V1.4. (Olympus, Japan). For scanning electron microscopy (SEM), cells were prepared using previously published protocol [41]. In brief, cells were chemically fixed (2.5% glutaraldehyde in f/2 medium, overnight at 4 °C) and then rinsed with f/2 medium, and the cell suspension was placed on poly-L-lysine coated coverslips. Post-fixation was performed using 1% OsO_4_ for 2 h at room temperature. After washing in f/2 medium, specimens were dehydrated with a graded series of acetone (30%–100%) for 15 min at each step, critical point dried (CPD2 by Pelco, Fresno, CA, USA), and gold coated using a sputter coater Baltec SCD 050. The samples were examined by FE-SEM JSM 7401-F (JEOL.; Tokyo, Japan) at an accelerating voltage of 3 kV using the GB-low mode.

### 2.3. Transmission Electron Microscopy

Cells were centrifuged (3000 rpm) for 10 min, and the compact pellet was directly loaded into high-pressure freezing (HPF) specimen carriers (1.2 mm in diameter, Leica, Vienna, Austria) or optionally immersed in 20% bovine serum albumin/f/2 medium for 30 min before freezing. Specimens were frozen using a high-pressure freezer (EM Pact, Leica, Germany) and placed in freeze substitution medium containing 2% OsO_4_ in 100% acetone pre-cooled to −90 °C. Freeze substitution was performed following the protocol described previously [41]. Specimens were rinsed three times with water-free acetone and gradually infiltrated with 25%, 50%, and 75% low-viscosity Spurr resin (SPI Supplies, West Chester, PA, USA) in acetone for 3 h at each step. Cells partially released from the HPF carriers were placed in microtubes. Tubes were submersed in a container filled with 2 L of water, and the specimens were irradiated in a microwave oven for 30 s at 80 W three times at each resin infiltration step. After overnight incubation in 100% Spurr, the material was embedded in fresh resin and polymerized at 60 °C for 48 h. Ultrathin sections were cut using an ultramicrotome (UCT.; Leica), collected on formwar-coated grids, contrasted in ethanolic uranyl acetate and lead citrate, and observed with a JEOL 1010 TEM (JEOL) at an acceleration voltage of 80 kV. Images were captured using a Mega View III camera (SIS.; Germany).

### 2.4. Confocal Microscopy

Two fluorescent dyes with a different affinity to color nuclear DNA (Hoechst 33342, Thermo-Fisher) and mitochondrial membranes (MitoTracker Orange CM-H2TMRos, Thermo-Fisher) were chosen to stain live cells. One to two milliliters of cultured cells were gently spun for 10 min at 5000 rcf; the supernatant was removed and replaced by isotonic sea salt solution (33.3 g/L). Both dyes were added into the sea salt solution at a concentration of 1:1000, and tubes were placed in the dark for 30 min. Slides for the confocal microscopy were prepared by placing stained cells on Superfrost Plus slides (Thermo-Fisher) to allow them to adhere to the slide surface. Excess dye was washed out by the sea salt solution to avoid cell disruption due to osmotic stress. Slides were then submerged in 4% paraformaldehyde (Sigma) for 30 min, using the sea salt solution to dissolve the paraformaldehyde. After fixing cells in paraformaldehyde, slides were washed in the sea salt solution for 10 min and mounted by ProLong Gold antifade reagent (Thermo-Fisher). Slides were observed using the Olympus FluoView FV1000 confocal microscope equipped with a multi-line argon laser and a mercury arc lamp. Fluorescence was visualized upon exposure of the slides to filtered light. For Hoechst 33342, we used excitation at 405 nm and emission was filtered using a dichroic mirror SDM560 and a barrier filter of 425–475 nm; for MitoTracker Orange CM-H2TMRos, we used excitation at 559 nm, and emission was filtered with SDM640 and a barrier filter of 575–620 nm. Fourteen layers at 0.35 μm per slice were collected for each Z stack, which is the optimal value for the objective lens (100×) used. Obtained images were further analyzed by the real-time interactive data visualization software IMARIS Scientific 3D/4D x64 6.2.1 (Bitplane, Belfast, UK) to create the 3D models of stained organelles.

### 2.5. DNA Isolation and rDNA PCR

Genomic DNA was isolated using the DNeasy Tissue kit (Qiagen), following the manufacturer´s instructions; DNA was quantified using a NanoDrop 1000 spectrophotometer (Thermo-Fisher). Ribosomal DNA was amplified using the AccuPrime high-fidelity Taq polymerase (Thermo-Fisher) with primers eurib_F (5′-AACCTGGTTGATCCTGCCAGT-3′) and eurib_R (5′-TGATCCTTCTGCAGGTTCACCTAC-3′) [42]. The cycling conditions of the PCR were as follows: 2 min at 94 °C.; and 30×[20 s at 94 °C.; 20 s at 61 °C.; 2 min at 68 °C]. Amplicons were verified on 1% agarose gel stained by GelRed (Biotium, Hayward, CA, USA) and cloned into the pGEM-T Easy vector (Promega, USA). Plasmids from randomly chosen colonies were purified and sequenced using M13 primers by GATC Biotech (Konstanz, Germany). Eight variant sequences of *C. marina* were obtained. Preparation of the genomic DNA library was carried out by the commercial company Macrogen (Seoul, Korea), as well as all the post DNA isolation steps.

### 2.6. Mitochondrial Genome Assembly and Analysis

Raw reads were quality- and adapter-trimmed using trimmomatic v0.36 [43]. Bacterial reads were removed by the BlobTools workflow [44] searched against the NCBI RefSeq database (accessed April 6, 2018). Reads were assembled using SPAdes v3.11.1 [45]. The completeness of the assembly was assessed by searching for conserved eukaryotic single-copy orthologs with BUSCO2 [46]. The mitochondrial genome contig was identified by the similarity with the *C. roenbergensis* mt DNA (GenBank: AF193903) and annotated using MFannot (http://megasun.bch.umontreal.ca/cgi-bin/mfannot/mfannotInterface.pl, last accessed 18-05-2018), and tRNA genes were predicted by tRNAscan-SE v2.0.0 [47], followed by manual curation. Furthermore, predicted gene borders and annotations were individually checked using BLAST against the NCBI database. AUG and alternative triplets known to serve as mt initiation codons (AUA in *nad7* and AUU in *rpl5* [48]) were considered as the starting points of translation, and only tRNAs on the transcribed strand were kept. Similarly, all other stramenopile mt genomes listed in Supplementary Tables S1 and S2 used for comparison were re-annotated with the up-to-date database of MFannot to allow a comparison of the gene content. No systematic improvement of annotation was attempted in these sequences. The circular map of the *C. marina* mt genome was generated with OGDRAW [49].

### 2.7. Phylogenetic Analyses

The obtained sequences were compared by a BLAST search with those available from the NCBI database to find the closest related taxa. Sequences of *C. marina* were manually inspected for errors and assembled using Geneious v11.1.4 [50]. Sequences of known bicosoecids and other stramenopiles were added to the final dataset, with apicomplexans and chlorophytes used as an outgroup. Sequences were aligned in the software MAFFT [51] with the default settings; the final alignment (1609 bp) was edited in Geneious v11.1.4. The best model of nucleotide substitution was chosen using jModelTest-2.1.4 [52]. Phylogenetic analyses were run under Bayesian Inference (BI) in the software MrBayes-3.2.6. [53]. BI runs were performed for 5 million generations, with four chains and four independent runs under the GTR+G+I model of molecular evolution. The coherence of each run was checked in TRACER v1.5 [54], and the estimated burn-in was 450,000 runs. The Maximum Likelihood (ML) tree was generated by the software PHYML [55], with the reliability of branching patterns within trees determined with 1000 resampling steps using the bootstrap method. The final tree containing both types of support (posterior probability and bootstrap) was visualized and edited in FigTree v1.4.3. [56].

## 3. Results

### 3.1. Cultivation

*C. marina* was isolated from a mixed environmental sample of algae living on the surface of a rock collected in Kvernesfjorden bay, Norway (see Material and Methods), in which nanoflagellates were observed in the mucilage created by a pelagophyte alga (which we were not able to identify to a species level). The flagellate was extracted by placing culture into the darkness to eradicate the alga; the remaining ciliates and dinoflagellates were removed from the culture by using a variety of low-dose antibiotics and by culture dilution to get monoclonal colonies. F/2 medium was initially supplemented with TSB and SA.; which allowed the growth of various bacteria in the isolate. After some rounds of sub-culturing, the bacterial community was phenotypically homogenous, consisting only of bacteria identified by its 16S rDNA sequences as *Sphingopyxis litoris* strain Th8 (KC756877). These co-isolated bacteria were kept in co-cultures with *C. marina* as its food source.

### 3.2. Cell Morphology

**Figure 1:**
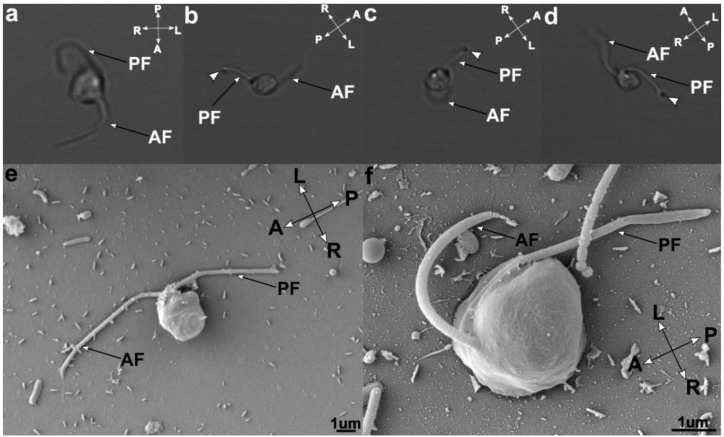
Cell appearance as observed using light microscopy (**a**,**b**,**c**,**d**) and scanning electron microscopy (SEM) (**e**,**f**). Light microscopy pictures show the attachment of the posterior flagellae (PF) to the surface (arrow) and the movement of the anterior flagellum (AF). SEM pictures (**e**,**f**) of *Cafileria marina* capturing the smooth surface of the cell wall and flagellae positions. The cell orientation was established arbitrarily by declaring the swimming direction to be forward and as follows: A: anterior, P: posterior, L: left, and R: right.

The orientation of the cell was determined by the swimming direction being forward. Cells of *C. marina* have a rounded shape on the right side of the cell and a flat shape on the left side, resembling the shape of a capital “D”; the flagellae sub-apically emerge from a dent on the ventral side. The nanoflagellate is 3–4 µm wide and 5–6 µm long; the surface of the whole cell is smooth without any structural features that are visible by light or scanning electron microscopy (Figure 1a,b). *C. marina* has smooth anterior and posterior flagellae of equal length, approximately 1.5–2 times the length of the body. *C. marina* exhibits a tumbling movement. The anterior flagellum is free and sweeping, while the posterior one is used as an anchor and is attached to the surface. *C. marina* is a constant feeder, with the cytostome permanently formed. Spores or resting stages were never observed during our work with the organism. 

### 3.3. Ultrastructure

**Figure 2:**
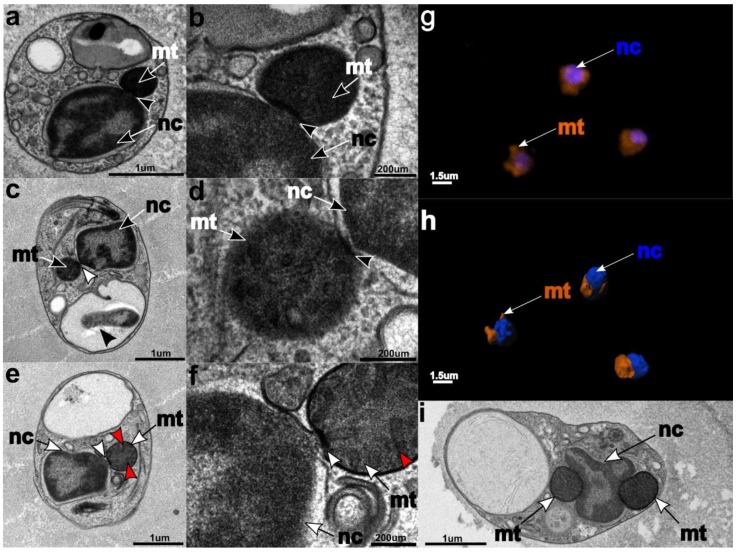
Connections between the nucleus (nc) and the mitochondria (mt) were documented several times independently (white arrows) (**a**,**b**,**c**,**d**,**e**,**f**). The bicosoecids’ mt has tubular cristae (**e**,**f**: red arrows). The connection of organelles was also observed using fluorescent staining of nc and mt and using confocal microscopy (**g**). Confocal Z-stacks were transformed into the 3D model (**h**) and mt appear in a location close to the nc. Mt can vary in shape and size, ranging from a simple spherical shape to a horseshoe shape, which is usually “hugging/wrapping” the whole nc (**i**). In the panel (**c**), devoured bacteria are visible in the food vacuole (black arrow).

**Figure 3:**
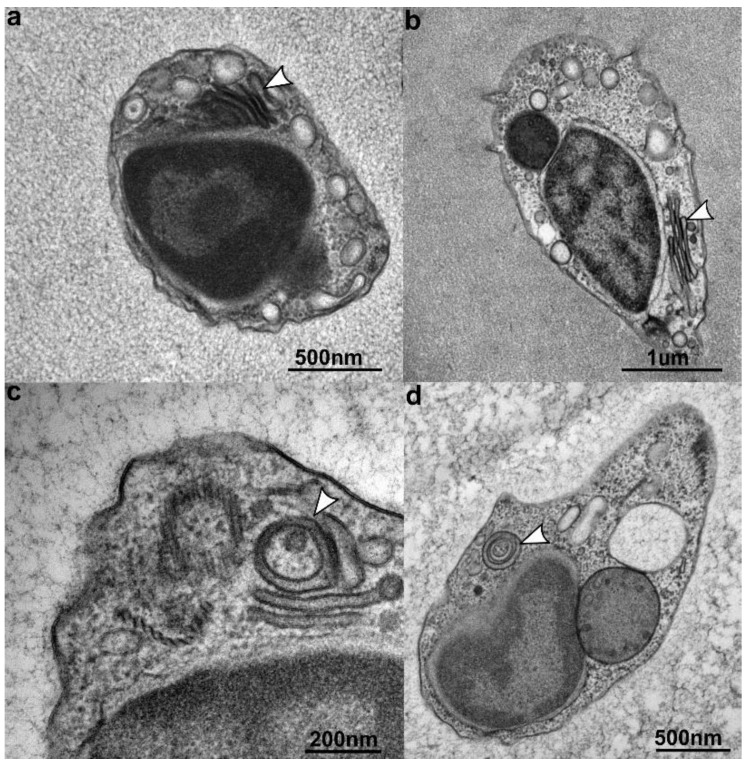
The Golgi apparatus changes shape during the cell cycle (arrow). The Golgi progressively changes from flat-shaped cisternae (**a**,**b**) stacked on top of each other to a rounded, circular, almost dish-like, shape (**c**,**d**).

The nucleus and mitochondria localize in the anterior part of the cell (Figure 2). The nucleus has a slightly elongated ovoid shape. The shape of the mitochondria varies from rod-shaped to horseshoe-shaped, and they are always found in close proximity to the nucleus (Figure 2). In young cultures (two weeks and younger), “junctions” are visible between the nucleus and mitochondria, where the mitochondrial and nuclear membranes appear partially dissolved and retreated to create an electron-dense disc between these compartments (Figure 2a–e). This phenomenon was observed during the early life stage of the flagellate. The mitochondrial cristae are tubular (Figure 2e,f), like in other stramenopile mitochondria. The Golgi is situated in the anterior part of the cell, with the cisternae aligning parallel to the nuclear surface. The number of cisternae varies between four and five (Figure 3). The shape of the Golgi apparatus changes from flat-stacked (Figure 3a,b) to rounded cisternae (Figure 3c,d), most likely depending on metabolic demands and the phase of the cell cycle [57,58]. In a more detailed view, the flat cisternae were seen to be curving inside and in some cases, created hollowed rounded shapes. Numerous small vesicles were scattered in the cytosol, while food vacuoles were placed in the posterior part and were considerably larger, taking up almost one third of the cell volume. Some of the food vacuoles contained intact or partially digested bacteria, which corroborates that *C. marina* is bacterivorous.

### 3.4. Flagellar Apparatus

**Figure 4:**
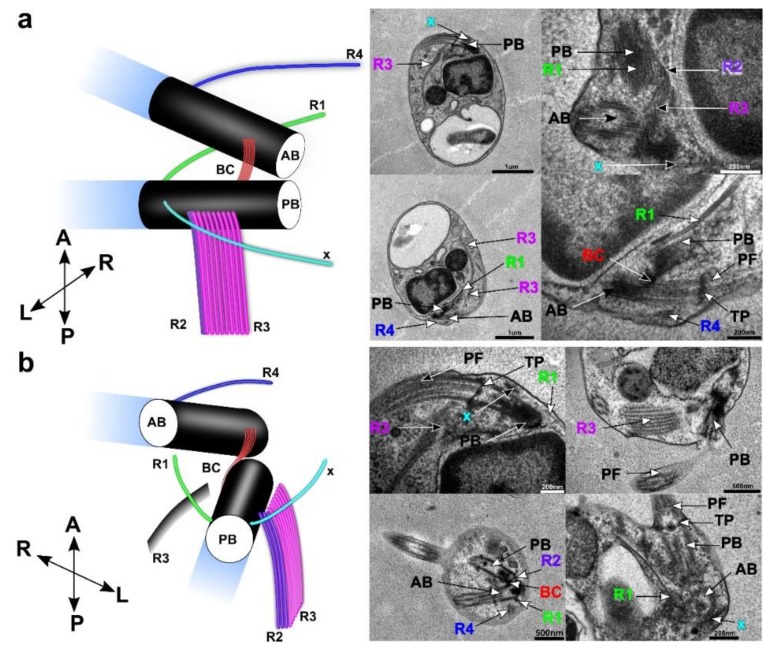
The flagellar apparatus (FA) was reconstructed based on TEM pictures; 3D models of the side view (**a**) and front view (**b**) were created. Pictures corresponding to the different view angles were added with morphological structures marked in the pictures. The whole FA comprised of the anterior and posterior basal body (AB and PB.; respectively), the connection between basal bodies (BC), four microtubular roots (R1–R4), and the multi-microtubular structure “abc”. In TEM pictures, transition plates (TP) of the anterior and the posterior flagella (AF and BF.; respectively) can also be seen. The cell orientation is shown in the left corner of the drawing: A: anterior, P: posterior, L: left, and R: right. Microtubular roots R1–R4 are the main structures forming the FA and supporting the feeding basket of *C. marina*; the R3 root is the defining morphological structure for Bicosoecida and consists of eight microtubules; the R2 root consists of three microtubules; and the remaining roots R1, R4, and ‘x’ consist of one microtubule. The number and angle of microtubules of the each root and their presence/absence are species-specific, so these structures are utilized as species-determining features.

The flagellar apparatus of *C. marina* was inspected from different angles using TEM cross sections (Figure 4). Basal bodies (anterior basal body: AB.; posterior basal body: PB) were situated in the anterior region of the cell, usually arranged at an acute (45°) angle to each other (Figure 4a). Both basal bodies were connected with one striated fiber extended between their proximal ends. Each flagellum consisted of a 9+2 axoneme, and both flagellae lacked any paraxial inclusions or flagellar hairs (Figure 1). The flagellae (anterior flagellum: AF.; posterior flagellum: PF) were connected to the corresponding basal body via a concave transition plate, from which the two inner axonemes appeared to grow. The basal bodies were placed in the anterior part of the cell and connected with electron-dense fibers (Figure 4a,b: BC). Four roots (R1–4) and additional tubular structures were discovered in the TEM pictures. Because R3 consists of eight microtubules and passes along the anterior part of the cell, it is also the most visible and distinctive character of the flagellar apparatus. Root R3 starts under the proximal ends of PB. Tubules follow the perimeter of the cell in the anterior part, and the whole structure forms a loop around the cytostome. An additional tubular structure, rootlet R2, has three microtubules horizontally positioned next to each other and at right angles to both PB and R3. Therefore, the R2 and R3 tubules are arranged in an ‘L’ shape near the proximal end of PB.; where both sets nucleate. The R2 rootlet goes alongside the frontal part of the cell, initially copying the direction of R3 tubules, but then R2 splits and follows the cell membrane further down to the caudal end. The remaining roots (R1 and R4) all consist of single microtubules; R1 starts close to the distal end of PB and passes towards the left side of the cell. Similar to the R1 root, R4 follows the same direction, but starts at the distal end of AB. The last microtubular structure ‘x’ starts close to the right distal ends of both flagella and moves down along the right side of the cell. 

### 3.5. Phylogenetic Analysis

**Figure 5:**
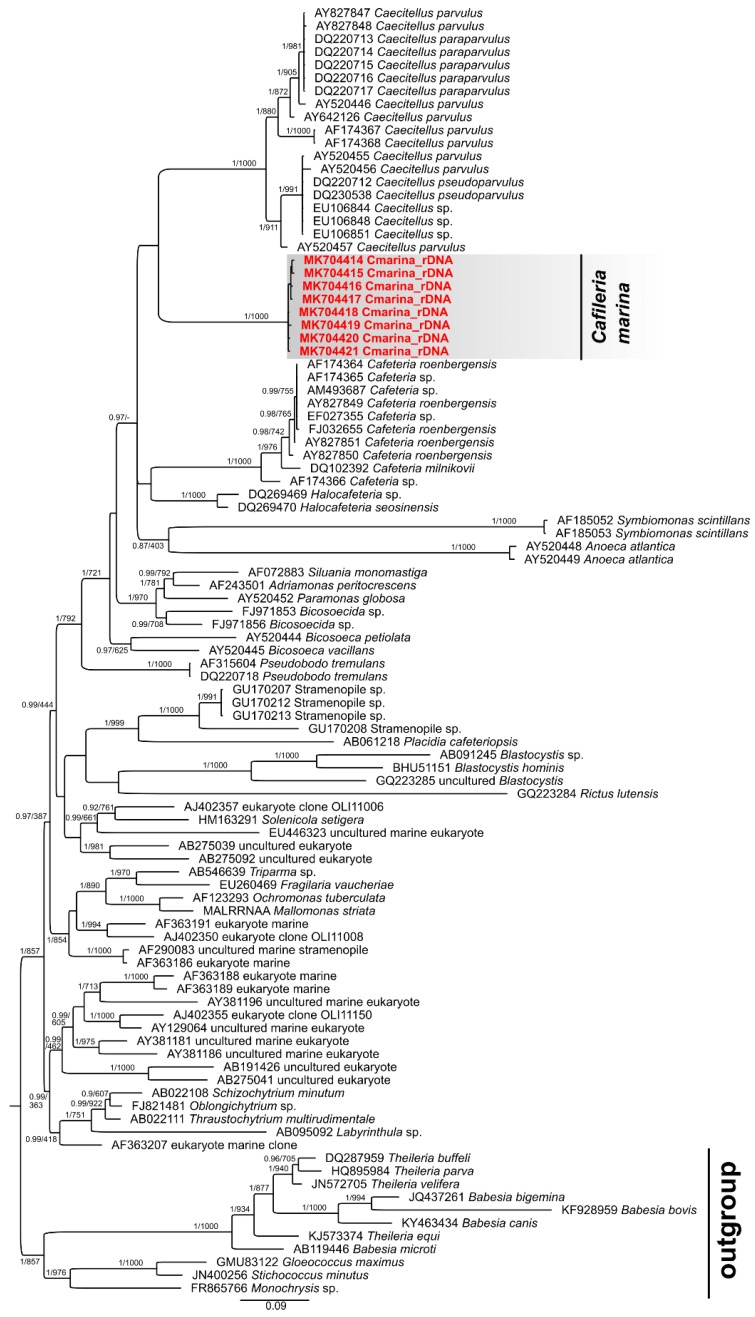
Phylogenetic tree based on 18S sequences of bicosoecids and closely related representatives of Stramenopiles with the final length of alignment of 1609 bp. The presented tree was computed by the software MrBayes. Sequences of apicomplexans were used as an outgroup to root the final tree. Statistical support for each branch was obtained from the Bayesian posterior probability and maximum-likelihood bootstrap. Posterior probabilities lower than 0.86 are not shown in the figure.

Our 18S sequential data were joined with sequences from other bicosoecids found at NCBI GenBank^TM^ and apicomplexans used as an outgroup. We obtained eight rDNA sequences (7 amplicons 1753 bp, one partial sequence) from a mono-eukaryotic culture of *C. marina*, which were deposited in GenBank^TM^ under accession numbers MK704414-21. Both analyses provided very similar results, so only one BI tree is presented with posterior probabilities (PP) and bootstrap branch supports (Figure 5 and Figure S1). All these sequences clustered within the bicosoecid clade and formed an isolated group with *Caecitellus parvulus* as the closest related taxon (Figure 5). The rDNA sequences of *C. marina* differ by, at most, 13 bp (0.74% difference), where some alterations might have been caused by amplification or sequencing errors. The average difference between *C. marina* and *Caecitellus* rDNA sequences is 17.19%, well exceeding the intra-genus variability of *Caecitellus* rDNA sequences (7.19% difference). Interestingly, the sequence of *Rictus lutensis* clustered with *Blastocystis* sequences in both types of analyses (BI and ML); however, with a somewhat lower branch support in the former case, suggesting a long branch attraction artifact. Our analyses suggest that there is a diversity of undescribed bicosoecid sequences more distantly, but robustly, related to known species. *C. marina* and *Halocafeteria* rDNA sequences differ in a similar range (14.01%). We consider these differences large enough to delimit a new genus named *Cafileria*. 

### 3.6. Mitochondrial Genome

The assembled reads yielded 1613 contigs, with the best blast hits among heterotrophic stramenopiles (oomycetes, *Blastocystis*, *Cafeteria*). Due to the low genome coverage of the assembly, as assessed using BUSCO2 [46], contigs other than the mt genome contigs were not analyzed in this study, with the exception of a screen for the possible presence of a plastid (due to the ongoing discussions about the distribution of plastids of red algal origin in different groups of algae, see introduction). None of the genome contigs had similarities to known plastid genomes. Furthermore, screening of the predicted proteome from the contigs with the plastid protein prediction tool AFAFind [59] (utilizing the tool SignalP 3.0 NN [60]) resulted in a very low number of proteins with positive prediction results, which can be explained by the false positive rate of ASAFind. Furthermore, during our cultivation and microscopy work with *C. marina*, we never observed a change in phenotype between cells cultured in the dark or in the light. We therefore think that *C. marina* does not have a plastid.

Among the contigs, NODE_11 showed overall similarity to mt genomes, most notably that of *C. roenbergensis*. The phylogenetic analysis placed *cox1* as the NODE_11 sister to *C. roenbergensis* (Figure S2). NODE_11 was therefore identified as the mt genome of *C. marina*. The, *C. marina* mt genome is circular-mapping and 42,797 bp long, with a GC content of 21.3%. While the size and the gene content are similar to other heterotrophic stramenopile mt genomes, the GC content is lower (Table S1), second only to the genome of the structurally-reduced mt-related organelle of *Blastocystis* (Table S2). The single *rns* and *rnl* genes for the mt rDNA are organized in tandem. The genome of *C. marina* encodes tRNAs cognate for most amino acids, except for Thr, Ala, and Gly (Figure S2). These tRNAs are frequently present in the mt proteins (4.8% for Ala, 6.4% for Gly, and 5.9% for Thr). A suppressor tRNA cognate to the UGA stop codon is also present.

**Figure 6:**
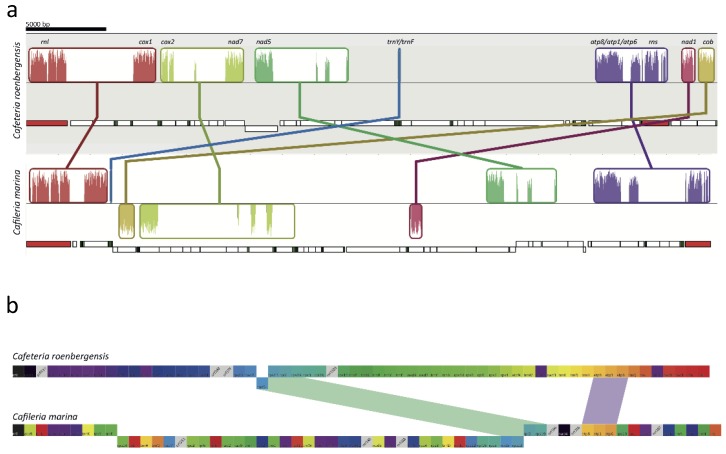
Collinearity of bicosoecid mitochondrial genomes. Panel (**a**) Very low level of collinearity on the genomic scale (as determined by Mauve) was observed among *Cafeteria roenbergensis* and *Cafileria marina*. Genes encoded by the plus and minus strand are depicted above or below the line representing the mitochondrial genomic sequence. Ribosomal, tRNA.; and protein-coding genes are schematically shown in red, green, and white, respectively. Gene-level collinearity Panel (**b**) is also very low, corroborating the numerous genomic rearrangements seen on the genomic level.

The genetic code is of type 4, with UGA coding for Trp and UAA/UAG serving as translational stops. In most cases, AUG is the initiation codon, but AUA and AUU are putatively used in *nad7* and *rpl5*, respectively. Protein-coding genes encode subunits of the respiratory complexes I.; III.; and IV.; and ATP synthase, and subunits of the ribosomal LSU and SSU. Except for the *C. marina rpl5*, the gene set is conserved in the two bicosoecids. The, *C. marina nad11* gene has a 426-bp extension at its 3’-end (compared to *Cafeteria*) that encodes a molybdopterin oxidoreductase 4Fe-4S domain. In most stramenopiles (but not in Chrysophyceae and Oomycetes), *nad11* is split into two independently translated ORFs [61]. *C. marina* is so far the only species known to encode the 4Fe-4S domain with the N-terminal ferredoxin-type NADH/ubiquinone oxidoreductase module rather than the C-terminal molybdopterin domains, adding to the list of evolutionary splits of *nad11* in stramenopiles [61]. The functional consequences of this modular arrangement of NAD11 are unclear. In addition to annotateable mt genes in *C. marina*, six hypothetical ORFs have no orthologs retrieved by a BLAST search, while the mt genome of *Cafeteria* shows four such regions. We could not find any group I or group II introns in the mt genome. Despite the quite conserved gene content in the mitochondria of bicosoecids, their order is highly rearranged in *C. marina* when compared to *Cafeteria* (Figure S2) and other heterotrophic stramenopiles (Figure 6). Alignment of *C. marina* and *C. roenbergensis* mt genomes revealed seven regions of higher similarity, most of which were limited to single genes. Only two gene clusters appear to share the same order in the two species (*atp8-atp1-atp6* and *nad5-rps13-rpl2-rps19*). Due to the scarce sampling of heterotrophic stramenopile mt genomes, it is not possible to decide which of the species is closer to the ancestral gene architecture.

## 4. Discussion

We conducted a thorough morphological, ultrastructural, and molecular examination of the novel marine heterotrophic nanoflagellate *C. marina.* It feeds on bacteria phagotrophically and belongs to the bicosoecids. Morphological and ultrastructural features typically described for bicosoecid cells—two flagella, the flagellar apparatus, microtubular root R3, and the tubular cristae in mitochondria [7,35,36]—were observed in *C. marina*. The bicosoecid lives and glides through the mucilage produced by a pelagophyte alga, with which it is in close association. A characteristic gliding/tumbling movement, with the anterior flagellum free and sweeping, while the posterior one was used as an anchor attached to the surface, was observed during cultivation. This type of movement has also been described for other bacteria-ingesting bicosoecids [62,63]. According to our observations, *C. marina* is a permanent feeder. Although the cytostome has not been seen in EM pictures, we assume that it is permanently present; firstly, due to the presence of the bacteria in the food vacuole (Figure 2c black arrow), and secondly, due to the similarity of the life style (bacteriovory in marine sediments?) of *Caecitellus parvulus* [64] and *Rictus lutensis* [5], which are two related bicosoecids with cytostomes. This supposition is corroborated by the presence of R3 and ‘x’, which are structures typically associated with support of the feeding lip and the starting point of the cytostome [37]. Available studies of bicosoecids have shown that they have four microtubular roots [37,65,66]. For the description of ultrastructural characters, we used previously established terminology [37]. The flagellar apparatus of *C. marina* is similar to *C. parvulus* and *R. lutensis* [5]; however, some tubular features are different/missing. The same ultrastructural characters of the flagellar apparatus are found in *C. marina*, *C. parvulus*, *R. lutensis*, and *C. roenbergensis*; however, the number of microtubules for roots differs from the above named species. The most visible difference is in the R3 root: *C. parvulus* has ~8–24 microtubules; *R. lutensis* has ~4–50 microtubules; and *C. roenbergensis* and *C. marina* have 8 microtubules. Some of the species also exhibit other unique features, e.g., *R. lutensis* has two additional microtubular structures ‘x’ and ‘S’, both of which are single-microtubule structures, whilst *C. roenbergensis* is so far the only species with secondary cytoskeletal microtubules on the R4 root (see Table 1 for details). The number of microtubules per root is thus unique in *C. marina*.

A peculiar major ultrastructural phenomenon of *C. marina* is the tight connection of the mitochondria and nucleus in young cultures (Figure 2). Although the clustering of mitochondria near the nucleus was documented for young rat heart cells [67], endothelial cells of pulmonary arteries in mammalian cells [68], mitochondria located in neurons [69], and in thymocytes and leukemia cell lines [70], a full conjunction of these two compartments, as seen in Figure 2, has not yet been described. This organellar conjunction might be parallel to the known tethering and synergy between the mitochondria and endoplasmic reticulum (ER) [71,72]. Their connection is involved in the regulation of lipid synthesis, Ca^2+^ signaling, and transport from ER to mitochondria [71,72,73]. The bonding between the two compartments directly affects the mitochondria biogenesis [74]. According to previous studies and results, we formed five hypotheses to explain this peculiarity: (i) The proximity of organelles might reflect intense ATP/ADP exchange between the mitochondria and the nucleus, via energy-dependent direct transport. The junction could serve as an energy "bridge" to cover the high energy demand of the nucleus during rapid cell growth and division [67,75]. A similar provision of energy has been described for multiple cell lines/types, e.g., cardiac cells [67] or endothelial cells of pulmonary arteries [68]; (ii) the connection might facilitate the direct transport of tRNAs that are not encoded by the mt genome, but are required for mitochondrial translation. This would be similar to the situation in *Trypanosoma brucei*, in which all mitochondrial tRNAs are encoded in the nucleus and are actively transported into the mitochondrion [76,77]. In the case of *C. marina*, alanine, glycine, and threonine (the amino acids carried by the exclusively nuclear-encoded tRNAs tRNA^Ala^, tRNA^Gly^, and tRNA^Thr^) are quite abundant (4.8%, 6.4%, and 5.9% of the predicted proteome, respectively) in the mitochondria-encoded proteins, hence the corresponding tRNAs must be imported into the mitochondrion; (iii) we should also take into consideration the hypothetical direct export of mRNA from the nucleus to the mitochondria as a template for the mitochondrial protein translation. Transport of mRNAs encoding mitochondrial proteins to the mitochondrial membrane and their cytosolic transcription is, in principle, known, but some mechanisms and associated processes are still not fully understood and explained [78,79]. Regarding the mRNA transport hypothesis, it is interesting to know that the sharing of mRNA molecules between cells has been described for placental cells under oxidative stress in the syncytial tissue; mRNAs transcribed by active cells can then diffuse freely throughout the syncytioplasm into transcriptionally-inactive cells of the syncytium [80]. However, currently, there is no known molecular machinery that can specifically recognize and transport mRNAs; (iv) the observed junction could also represent a physical connection of mitochondria and the nucleus during cell division to mechanically ensure equal segregation into the daughter cells. These cellular-level changes in organelle organization might also be linked to the variety of mitochondrial shapes, or be a special case of "kiss-and-run" mitochondrial motility dynamics, similar to the ones described by [81]; (v) last but not least, the observed junctions might simply be a consequence of the small cell size and efficient intracellular organization. Because of the small cell size, the ER is extremely reduced in *C. marina*. Since the nuclear envelope is continuous with the ER.; the observed junctions might also represent mitochondria-associated membranes. These are contact sites between ER and mitochondrial membranes [82], that in the case of *C. marina*, might reside directly at the outer side of the nuclear envelope.

In addition to the nucleus–mitochondria connections, we observed changes in the shape of the *C. marina* Golgi apparatus during the cell cycle (Figure 3). Interestingly, different forms and arrangements of Golgi cisternae seem to correlate with different cell cycle stages. A similar phenomenon was predicted for mammalian cells [58,83] and later observed in vivo [57]. While the flattening and curvature of the human Golgi has been associated with membrane curvature generators and changes in sphingomyelin metabolism [57], in the case of the described nanoflagellate, the mechanism remains elusive.

The performed phylogenetic and morphological analyses with the comparison to other studies showed that *C. parvulus* and the representatives of the family Cafeteriidae (*Cafeteria* sp. and *Cafeteria mylnikovii*) are the closest relatives to the novel bicosoecid species. Although the morphological features showed more similarities to *C. roenbergensis,* namely the microtubule number of individual roots, shown in Table 1, both types of molecular data (mt genome and 18S) share more similarities with *C. parvulus*. The results of our phylogenetic analyses showed that *C. marina* is not only a newly discovered species, but its divergence also qualifies it for placement in a new genus. Moreover, the already mentioned paraphyly of the genus *Cafeteria* in [37] and [26] was confirmed by our analyses. However, this assumption is only based on the position of a single sequence of *Pseudobodo tremulans* in [37] and of *Cafeteria* sp. in [26], and further investigation is thus needed to confirm this claim.

Besides the *C*. *roenbergensis* mt genome, there is no other larger-scale bicosoecid data available for comparison. Our analyses showed a similar composition of the *C. marina* mt genome to the *C. roenbergensis* mt genome; however, with a different gene arrangement. The features typical for all stramenopiles, the lack of tRNA^Thr^ and the presence of suppressor tRNA consanguineous to the UGA stop codon, were also found in the *C. roenbergensis* mt genome, but the lack of tRNA^Ala^ and tRNA^Gly^ had been unique to *C. marina* up to now.

To summarize our findings, phylogenetic and morphological data concordantly place *Cafileria marina* into the family Bicosoecidae (Stein, 1878), with enough diverging evidence from known taxa to propose *C. marina* as a new species and genus of this family.

### 4.1. Taxonomic Summary

*Cafileria marina* is a heterotrophic stramenopile feeding on bacteria and is described under the International Code of Zoological Nomenclature.

### 4.2. Cafileria n. gen.

Flattened pyriform cells with two naked flagella, displaying tumbling movement with one flagellum attached to the substrate and the other flagellum moving. They have no lorica, cell wall, or other surface structures, and feed on bacteria by phagotrophy.

### 4.3. Type species

Cafileria marina n. sp.

### 4.4. Type locality

A three-meter depth off the shore in Gaustad, Norway (Kvernesfjorden fjord, 62°59′07.9″N 7°19′17.4″E), was used in this study.

### 4.5. Cafileria marina n. sp.

#### 4.5.1. Description

Cells 3–4 µm wide and 5–6 µm long, with anterior and posterior flagella of equal length that were 1.5–2 times length of the body, were employed. The cells and flagella had a smooth surface. The flagellar apparatus consisted of four roots (R1–R4) that did not have any secondary cytoskeletal microtubules. 

#### 4.5.2. Etymology

*Cafileria marina* is named after the “kafilerie”, the Czech name of the place where the biomass of animal origin is dismantled and conditioned for the production of lipids, glue, and fertilizers. Therefore, we have seen a parallel with the feeding habits of the newly discovered nanoflagellate, which feasts on bacteria that recycle organic materials found in the biofilm mucillage that makes up their habitat. The species name *marina* stands for the marine origin of the species.

#### 4.5.3. Hapantotype

The designated hapantotype has been deposited as TEM and SEM specimens, and cryo-mixed cultures of nanoflagellate *C. marina* and bacteria *S. litoris* in the slide collection at the Biological Centre of the Czech Academy of Sciences, Institute of Parasitology, Ceske Budejovice, Czech Republic, under the accession number IP CAS Pro 59.

#### 4.5.4. Assignation

Eukaryota; Stramenopila; Bicosoecida.

## Figures and Tables

**Table 1:** Comparison of the *Cafileria marina* flagellar apparatus with closely related bicosoecids. The terminology was adopted from [30]. ‘S’ is the additional microtubular structure unique to *R. lutensis*, similar to ‘x’, which consists of a single microtubule, but lays on top of the R3 root and copies its shape.

Ultrastructure of the Flagellar Apparatus	R1			R2			R3			R4			Additional Microtubular Structures
	Present	Number of Microtubules	Original Name in the Publication	Present	Number of Microtubules	Original Name in the Publication	Present	Number of Microtubules	Original Name in the Publication	Present	Number of Microtubules	Original Name in the Publication	
Species and publication													
*Halocafeteria seosinensis*	Not confirmed			+	8		Not confirmed			Not confirmed			
Park et al., 2006 [7]													
*Cafeteria roenbergensis*	+	2	R4	+	3	abc	+	8		+	2	R1	x
O’Kelly and Patterson 1996 [35]										Secondary cytoskeletal microtubules			
*Rictus lutensis*	+	2	R4	+	3	abc	+	~4–50	R2	+	2	R3	x
Yubuki et al., 2010 [5]													S
*Caecitellus parvulus*	+	2	R1	+	3	abc	+	~8–24	R2	+	2	R1	x
O’Kelly and Nerad 1998 [64]													
*Cafileria marina*	+	1		+	3		+	8		+	1		x

## Supplementary Materials

Supplementary materials can be found at https://www.mdpi.com/2076-2607/7/8/240/s1.
