# Supplementary figures and images for: Morphology, Ultrastructure, and Mitochondrial Genome of the Marine Non-Photosynthetic Bicosoecid Cafileria marina Gen. et sp. nov
Source: Microorganisms. 2019 Aug 5;7(8):240. doi: 10.3390/microorganisms7080240 (PMC6723347; doi:10.3390/microorganisms7080240)

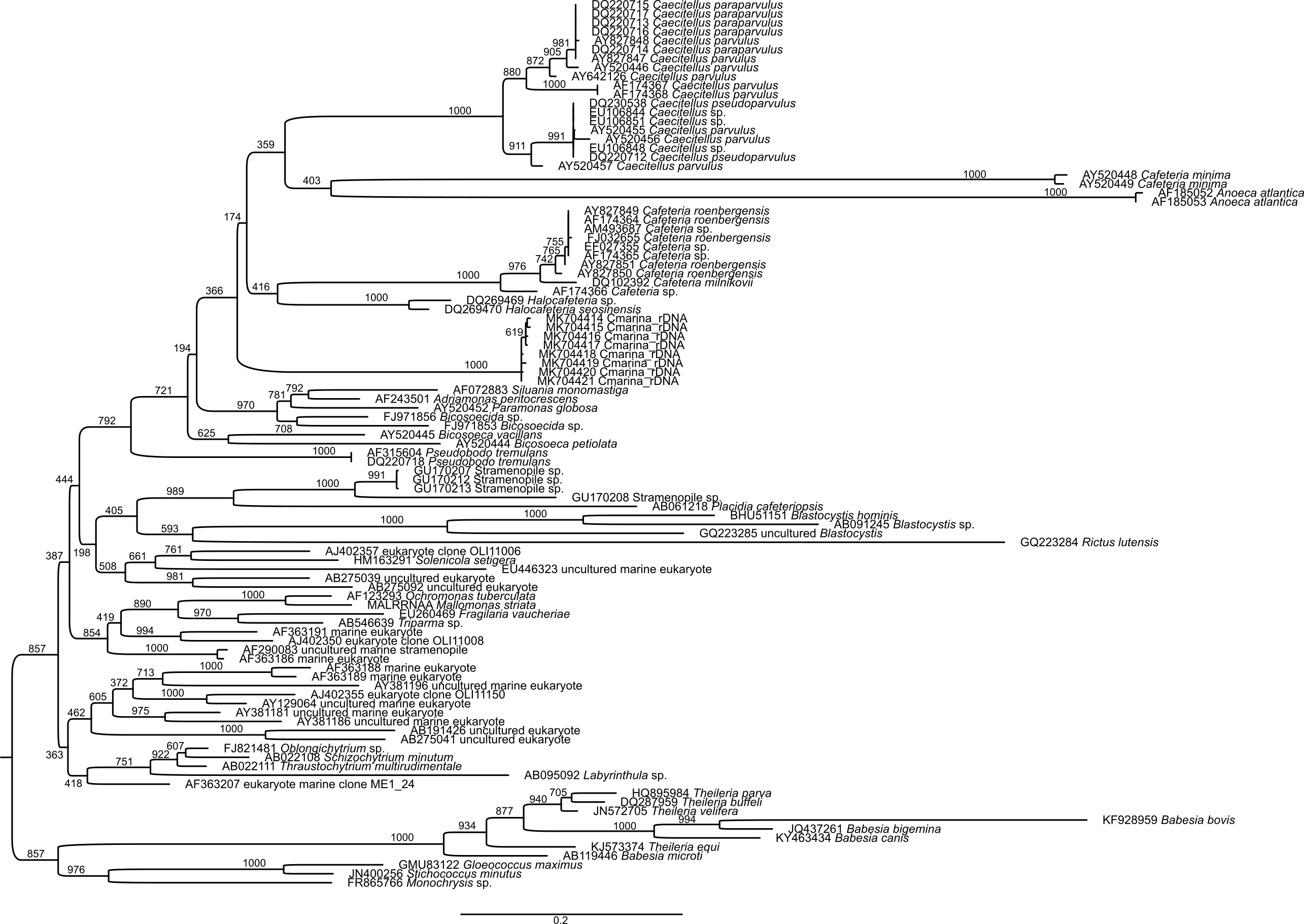

Supplement: Supplementary file 1 [file microorganisms-07-00240-s001.zip › Supplementary_data/Figure_S1.tiff]

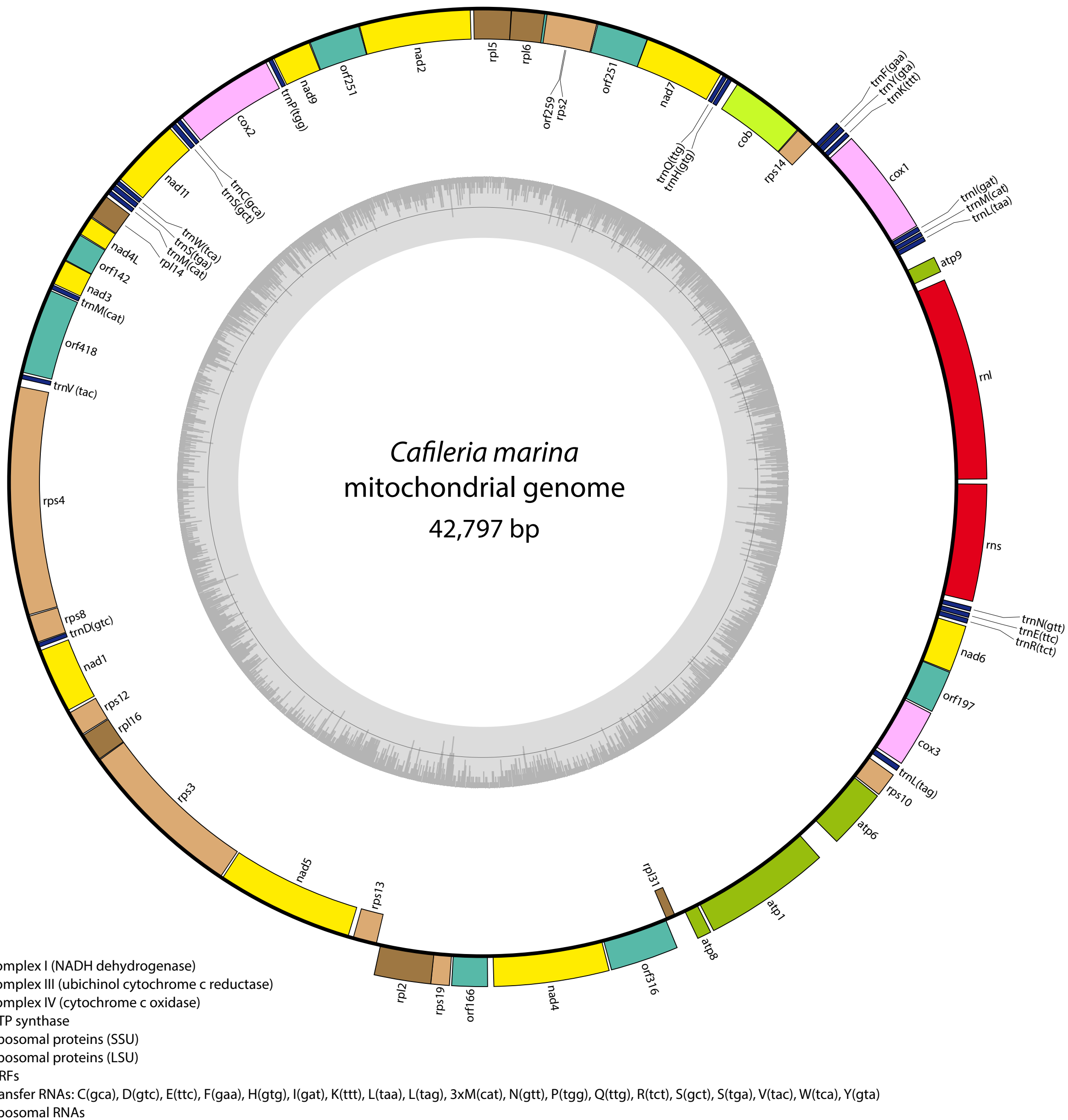

Supplement: Supplementary file 1 [file microorganisms-07-00240-s001.zip › Supplementary_data/Figure-S2.pdf]
